# Incomplete Evidence of Bone Density Normalization Following Long-Term Reproductive Hormone Treatment in Men With Hypogonadotropic Hypogonadism

**DOI:** 10.1210/clinem/dgaf488

**Published:** 2025-10-01

**Authors:** Nipun Lakshitha de Silva, Elizabeth Hyams, Bonnie Grant, Paras Dixit, Rajdeep Bassi, Paul Bassett, Alexander N Comninos, Channa N Jayasena

**Affiliations:** Section of Investigative Medicine, Imperial College London, Commonwealth Building, Hammersmith Hospital, London W12 0NN, UK; Department of Clinical Sciences, Faculty of Medicine, General Sir John Kotelawala Defence University, Ratmalana 10370, Sri Lanka; Section of Investigative Medicine, Imperial College London, Commonwealth Building, Hammersmith Hospital, London W12 0NN, UK; Section of Investigative Medicine, Imperial College London, Commonwealth Building, Hammersmith Hospital, London W12 0NN, UK; Section of Investigative Medicine, Imperial College London, Commonwealth Building, Hammersmith Hospital, London W12 0NN, UK; Section of Investigative Medicine, Imperial College London, Commonwealth Building, Hammersmith Hospital, London W12 0NN, UK; Statsconsultancy Ltd, Amersham, Bucks HP7 9EN, UK; Section of Investigative Medicine, Imperial College London, Commonwealth Building, Hammersmith Hospital, London W12 0NN, UK; Endocrine Bone Unit, Imperial College Healthcare NHS Trust, London W2 1NY, UK; Section of Investigative Medicine, Imperial College London, Commonwealth Building, Hammersmith Hospital, London W12 0NN, UK

**Keywords:** hypogonadotropic hypogonadism, Kallmann syndrome, osteoporosis, fractures, metabolic bone disease, bone mineral density

## Abstract

**Context:**

The prevalence and severity of low bone mineral density (BMD) in hypogonadotropic hypogonadism (HH), as well as the ability of reproductive hormone treatment to normalize BMD have not been investigated in large multicenter studies.

**Objective:**

We performed a systemic review and meta-analysis of several small, observational studies to investigate the effect of reproductive hormone treatment on BMD in men with HH compared with control groups where available.

**Methods:**

We searched OVID Medline, Embase, CINAHL, SCOPUS, Web of Science, and Cochrane Library for studies reporting BMD or fractures in men with HH (congenital [CHH] or acquired). Study selection and data extraction were performed using COVIDENCE and a prespecified tool. Results were summarized using descriptive statistics. Meta-analysis compared BMD in men with HH vs healthy controls. Meta-regression assessed relationships between treatment duration and BMD Z-scores against normative population data.

**Results:**

Of the 33 eligible studies, 24 included data specific to men with HH (n = 625). Men with HH had low lumbar spine (LS) and femoral neck BMD, improving with hormonal treatment. Meta-analysis of 5 studies found lower LS BMD in men with HH vs healthy controls (SMD −5.98; 95% CI; −11.5 to −0.47). Men with CHH may have persistently low BMD despite prolonged hormonal treatment. Higher BMD in HH was associated with younger age at treatment initiation, partial HH, and higher serum testosterone and estradiol concentrations. Fracture prevalence was high in the few studies systematically studying fractures as an outcome; in other studies, fractures were seldom reported.

**Conclusion:**

Men with HH have low BMD that improves with reproductive hormone treatment. However, current evidence suggests that incomplete BMD normalization may be common despite long-term reproductive hormone treatment in men with HH, particularly those with CHH.

Hypogonadotropic hypogonadism (HH) affects approximately 1 in 2000 men (1). HH may present prepubertally (congenital HH; CHH) or in postpubertal adults (acquired HH). Testosterone increases bone strength and density directly through its action on androgen receptors and indirectly after conversion to estradiol, which binds to estrogen receptors (2). Mechanistically, activation of androgen receptors in bone primarily stimulates osteoblast proliferation and inhibits osteoclast activity, while activation of estrogen receptors primarily inhibits osteoclast proliferation and promotes osteoclast apoptosis (3). Therefore, hypogonadism is a well-known cause of osteoporosis in men (3). Testosterone treatment, the standard treatment for HH, has beneficial effects on bone mineral density (BMD) in men with hypogonadism (4). The beneficial effects of testosterone treatment on bone mineralization have been studied within large randomized controlled studies (RCTs) of men with functional hypogonadism associated with older age or comorbidities although effects on fracture risk remain controversial (5, 6). However, it is unethical to conduct prolonged placebo-controlled RCTs in men with HH. Therefore, the effects of testosterone replacement therapy (TRT) on bone outcomes in HH have been studied only in modestly sized observational studies. Similarly, there is scarcity of bone data on other reproductive hormone replacement modalities (gonadotrophins and gonadotropin-releasing hormone pulse therapy) that stimulate endogenous testosterone secretion. In summary, the extent to which reproductive hormone therapies improve and normalize BMD in men with HH has not been conclusively studied to date.

This systematic review was conducted to investigate the following in men with HH: a) BMD compared with healthy eugonadal men or men with other relevant diseases; b) changes in BMD with reproductive hormone treatment; c) the extent to which BMD normalizes compared with the population with reproductive hormone treatment.

## Materials and Methods

We followed the Preferred Reporting Items for Systematic Reviews and Meta-Analyses (PRISMA) guidelines in this systematic review and meta-analysis. This systematic review was prospectively registered in PROSPERO (CRD42024569718).

### Search Strategy and Study Selection

We searched for studies reporting BMD or fracture outcomes in men with HH (either CHH or acquired) with or without a comparator group. The most common cause for acquired HH was pituitary pathology (eg, pituitary tumors or surgery). We excluded (1) studies of men with other causes of male hypogonadism such as testicular failure, functional hypogonadism (obesity, diabetes, severe energy deficit); (2) men with hypogonadism due to causes that can independently affect bone health and would confound the ability to assess testosterone effects (thalassemia, hereditary hemochromatosis, acromegaly, Cushing syndrome, prostate cancer); (3) studies of adolescents; (4) case reports, case series with fewer than 5 eligible patients, publications without an English full-text article and conference abstracts.

We searched OVID Medline, Embase, CINAHL, SCOPUS, Web of Science, and Cochrane Library from inception to July 11, 2024. We performed a key word search in Medline using the terms “*hypogonadotropic hypogonadism*” OR “*Kallmann syndrome*” that was combined with the key word search “*osteoporosis*” OR “*osteop?enia*” OR “fracture*” OR “*densitometry*” OR “*D?XA*” using the “AND” operator. A similar search was performed using Medical Subject Headings terms. The detailed search strategy for individual databases is provided in Supplementary File S1 (7). We also reviewed the references of selected full-text articles to identify additional studies.

Study selection and data extraction were performed using the COVIDENCE online platform (Veritas Health Innovation). Results of individual database searches were transferred to the COVIDENCE, and duplicates were removed. Titles and abstracts were reviewed independently for eligibility to screen full-text articles by 2 reviewers (R.B. and P.D.). The same reviewers reviewed the full-text articles for eligibility to extract data. Conflicts were resolved by a third reviewer (N.L. or E.H.).

### Data Extraction

Two reviewers (R.B. and P.D.) extracted data from selected full-text articles on relevant data extraction forms. All data extractions were verified by a second reviewer (N.L. or E.H.). We extracted data on the study design, participant characteristics, comparator characteristics (if available), treatment details, outcome measures, and results on predefined data extraction templates (Supplementary File 2 (8)).

Primary outcome measures of interest were fractures and low BMD. We defined low BMD as a Z-score less than or equal to −2 in any of the following sites using dual-energy x-ray absorptiometry: lumbar spine (LS), total hip (TH), femoral neck (FN), or distal forearm. If these were not reported, summary statistics of Z-score/ t-score or BMD were assessed.

### Quality Assessment

We used the Cochrane ROB tool for the risk of bias (ROB) of RCTs. For observational studies, we used the NIH quality assessment tool for observational cohort and cross-sectional studies (Supplementary File S3 (9)).

### Data Analysis

We summarized the reported BMD and fracture data using descriptive statistics. We performed meta-analyses to compare BMD in men with HH to healthy controls. In comparing men with HH to healthy controls, we performed sensitivity analyses depending on the men’s treatment status. We also performed a meta-analysis and a meta-regression of Z-scores of LS and FN BMD in men with HH, considering the treatment duration and patient group (CHH, acquired or mixed, ie, study cohort included both CHH and acquired HH) as covariates. Age was not included as a covariate, as Z-scores adjust for this. The meta-regression with treatment duration was limited to studies reporting patients with CHH due to the inadequate number of studies with acquired HH. A random-effects model was used due to the small number of studies included in the meta-analysis. I-squared (*I^2^*) statistics were used to assess study heterogeneity.

## Results

**Figure 1.**
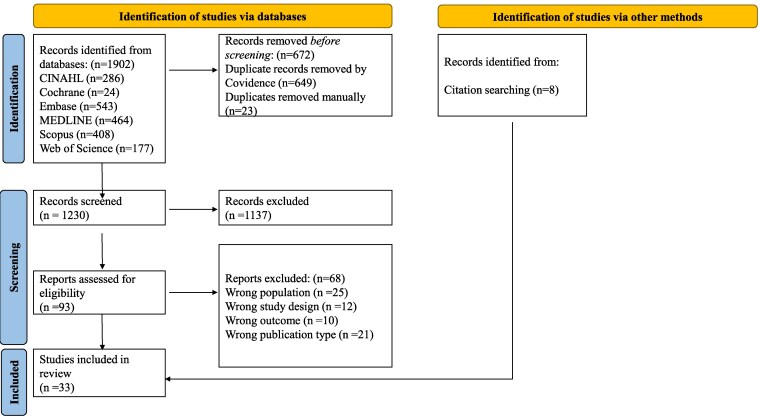
PRISMA flow diagram for the study selection. The database search identified 1902 studies. After removing duplicates, 1230 abstracts were screened and 93 were selected for full-text review. Twenty-five were included in the final review. Eight additional studies were identified through citation searching.

The database search yielded 1902 results, of which 1230 abstracts were reviewed for eligibility after removing duplicates (Fig. 1). We reviewed 93 full-text articles for eligibility, and 25 met the eligibility criteria. We additionally identified 8 studies from citation searching, resulting in 33 studies in the final review (10-42). Study characteristics are described in Supplementary File 4 (43). Although some studies we included were eligible based on reporting men with HH, the bone density results were mixed with another population such as women (22) or men with other causes of hypogonadism and were therefore excluded from further analysis (20, 26, 28, 29, 32, 35, 39, 40). A total of 625 men were included across the 24 analyzed studies.

### Quality of Evidence

The quality assessment of all the observational studies is summarized in Supplementary File 5 (44). Seventeen of the 33 studies had “fair” overall quality while 12 were rated “poor” and 3 were rated “good.” Not defining patient and control populations clearly, the lack of sample size estimation, not adjusting for confounders in the analysis, and the lack of blinding in the outcome assessment were the main contributing factors for lower ratings. The only eligible randomized controlled study was not included in the detailed synthesis since results were reported together with other causes of hypogonadism (29). However, this study was found to have “some concerns over the risk of bias,” according to our assessment using the ROB 2 tool.

### Bone Mineral Density in Men With Hypogonadotropic Hypogonadism Compared With Normative Population Data

To compare BMD in men with HH and population normative data, we performed meta-analyses and meta-regressions of Z-scores of LS and FN regions. Covariates within the analyses were classification of HH (acquired HH, CHH, and mixed, ie, both CHH and acquired HH) and treatment duration. T-scores were used in 3 studies when the Z-score was not reported, with the assumption that they would be similar to Z-scores in young people (10, 19, 24).

#### Lumbar spine

**Figure 2.**
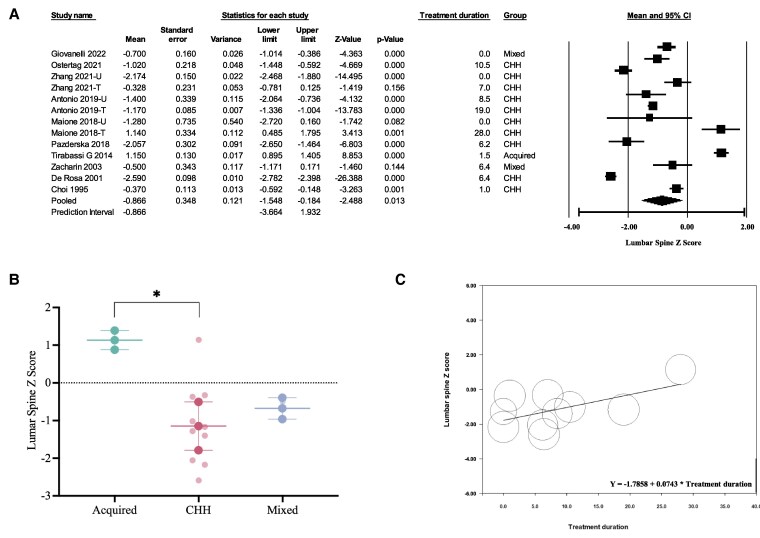
Meta-analysis and meta-regression of lumbar spine Z-scores in men with hypogonadotropic hypogonadism (HH). A, Meta-analysis of eligible studies reporting lumbar spine Z-scores with a pooled value of −0.866 (95% CI: −1.548 to −0.184). B, Meta-analysis by group showing individual study lumbar spine Z-score means (represented by pale dots) and the pooled mean with 95% CI. *P* values shown for meta-regression indicating differences between groups. C, Meta-regression of lumbar spine Z-score according to treatment duration in studies reporting data of men with congenital hypogonadotropic hypogonadism (CHH) only.

The pooled LS Z-score in men with HH was −0.87 (95% CI, −1.55 to −0.18) using data from all 10 studies with available data (10-14, 19, 24, 25, 30, 33) (Fig. 2A). The pooled mean LS Z-score for studies by group was 1.15 (95% CI, 0.90-1.41) for acquired HH, −1.13 (95% CI, −1.78 to −0.49) for CHH, and −0.66 (95% CI, −0.95 to −0.38) for mixed (ie, both CHH and acquired HH) (Fig. 2B). Meta-regression, considering the patient group and treatment duration, indicated lower Z-scores in men with CHH compared to the acquired HH group, with a coefficient of −2.28 (95% CI, −4.31 to −0.26; *P* = .027). A nonsignificant trend was observed toward increased Z-score with testosterone treatment duration with a mean increase of 0.07 per year (95% CI, −0.01 to 0.16; *P* = .094) in men with CHH (Fig. 2C). The *I^2^* statistic was 98%, suggesting that 98% of the variance in observed effects reflects heterogeneity between studies, rather than sampling error.

#### Femoral neck

**Figure 3.**
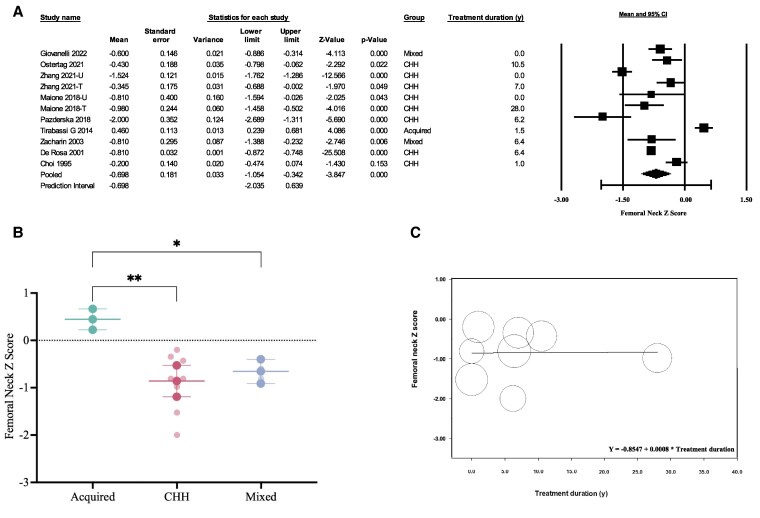
Meta-analysis and meta-regression of femoral neck Z-scores in men with hypogonadotropic hypogonadism (HH). A, Meta-analysis of eligible studies reporting femoral neck Z-scores with a pooled value of −0.698 (95% CI: −1.054 to −0.342). B, Meta-analysis by group showing individual study femoral neck Z-score means (represented by pale dots) and the pooled mean with 95% CI. *P* values shown for meta-regression indicating differences between groups. C, Meta-regression of femoral neck Z-score according to treatment duration in studies reporting data of men with congenital hypogonadotropic hypogonadism (CHH) only.

In 9 studies (11-14, 19, 24, 25, 30, 33) with available data on men with HH, the pooled FN Z-score was −0.70 (95% CI, −1.05 to −0.34) (Fig. 3A). The pooled FN Z-score for studies by group was 0.46 (95% CI, 0.24-0.68) for acquired, −0.85 (95% CI, −1.18 to −0.51) for CHH, and −0.64 (95% CI, −0.90 to −0.39) for mixed (Fig. 2B). Meta-regression found significantly lower FN Z-scores compared with the acquired HH group in both the CHH group (coefficient, −1.31; 95% CI, −2.21 to −0.40; *P* = .0049) and the mixed group (coefficient, −1.15; 95% CI, −2.22 to −0.08; *P* = .036). Treatment duration was not associated with FN Z-scores in men with CHH (*P* = .97) (Fig. 3C). The *I^2^* statistic was 95%, suggesting that 95% of the variance in observed effects reflects heterogeneity between studies, rather than sampling error.

### Comparing Bone Mineral Density in Men with Hypogonadotropic Hypogonadism Compared With Healthy Eugonadal Men

We identified several studies comparing bone density data in healthy eugonadal men compared with treated (13, 16, 25, 34, 37, 42), or untreated men with HH (16, 18, 21, 37, 38). Results of the studies comparing men with HH to healthy controls are summarized in Table 1.

**Table 1.** Summary of results in studies comparing men with hypogonadotropic hypogonadism to healthy controls or men with primary hypogonadism

Study ID	Outcome	Men with HH*^a^*	Healthy controls	Men with PH*^a^*	Significance
Giovanelli et al 2022 (11)	LS Z-score	−0.7 ± 1.1	—	−1.0 ± 1.4	.01
	FN Z-score	−0.6 ± 1.0	—	−0.7 ± 1.1	.07
	Vertebral fragility fractures	8/47	—	14/72	.6
	Other fragility fractures	2/47	—	2/72	.5
Ostertag et al 2021 (13)	LS BMD (g/cm^2^)	1.12 ± 0.19	1.23 ± 0.13	—	<.01
	LS Z-score	−1.02 ± 1.56	0.43 ± 1.11	—	<.0001
	FN BMD (g/cm^2^)	1.01 ± 0.18	1.12 ± 0.14	—	<.01
	FN Z-score	−0.43 ± 1.34	0.95 ± 1.08	—	<.0001
	TH BMD (g/cm^2^)	1.03 ± 0.20	1.13 ± 0.14	—	<.01
	TH Z-score	−0.43 ± 1.43	0.90 ± 1.13	—	<.0001
	Distal radius BMD (g/cm^2^)	0.48 ± 0.10	0.56 ± 0.06	—	<.0001
	Distal radius Z-score	−1.05 ± 1.93	0.76 ± 1.11	—	<.0001
	Number with Z-score < −2 in LS	16/51	0 (based on inclusion criteria)	—	NA
	Number with Z-score < −2 in distal radius	14/51	0 (based on inclusion criteria)	—	NA
	Number with fragility fractures	7/51	0 (based on inclusion criteria)	—	NA
Li et al 2015 (18)	Number of men with low BMD (Z-score < −2)	21/22	8/20	—	NR
	LS BMD (g/cm^2^)	0.91 ± 0.15	1.19 ± 0.09	—	<.001
	FN BMD(g/cm^2^)	0.802 ± 0.111	1.051 ± 0.055	—	<.001
	TH BMD (g/cm^2^)	0.838 ± 0.123	1.116 ± 0.069	—	<.001
Gioia et al 2014 (16)	BMD LS (g/cm^2^)	1.05 (0.9 to 1.13)	1.23 (0.97 to 1.5)	1.2 (0.95 to 1.35)	NS
	LS T-score	−1.3 (−2.3 to −0.7)	0.1 (−2.1 to 2.3)	−0.2 (−2.0 to 1.2)	<.01*^b^* < .05*^c^*
	LS Z-score	1.2 (−3.0; −0.2)	0.0 (−2.4 to 2.4)	−0.2 (−2.7 to 1.2)	NS
	FN BMD (g/cm^2^)	0.85 (0.73 to 0.94)	0.98 (0.74 to 1.56)	0.99 (0.82 to 1.17)	<0.01*^b^* < 0.01*^c^*
	FN T-score	−1.6 (−2.3 to −1.0)	−0.7 (−2.6 to 3.8)	−0.5 (−1.9 to 0.7)	<.01*^b^* < .01*^c^*
	FN Z-score	−1.05 (−1.8 to −0.4)	0.00 (−1.9 to 4.00)	−0.25 (−1.3 to 0.6)	<.01*^b^* < .05*^c^*
	TH BMD(g/cm^2^)	0.96 (0.81 to 1.02)	1.04 (0.84 to 1.57)	1.08 (0.78 to 1.24)	NS
	TH T-score	−0.9 (−1.6 to −0.4)	−0.4 (−1.9 to 3.7)	−0.1 (−1.1 to 1.1)	NS
	TH Z-score	−0.45 (−1.6 to 0.1)	0.0 (−1.6 to 3.7)	0.00 (−0.9 to 1.4)	NS
Bolu et al 2005 (21)	Vertebral BMD (g/cm^2^)	0.74 ± 0.11	0.99 ± 0.15	—	−.001
	Femur total BMD (g/cm^2^)	0.88 ± 0.13	1.05 ± 0.12	—	.001
	FN BMD (g/cm^2^)	0.86 ± 0.12	1.01 ± 0.13	—	.001
Schubert et al 2003 (27)	Trabecular (distal radius) Z-score	−1.52 ± 0.23	—	−0.87 ± 0.23	<.01
Zacharin et al 2003 (30)	LS BMD (g/cm^2^)	1.03 ± 0.16	—	1.01 ± 0.03	NS
	LS Z-score	−0.50 ± 1.37	—	−0.73 ± 0.28	NS
	LS T-score	−0.53 ± 1.40	—	−0.77 ± 0.27	NS
	FN BMD (g/cm^2^)	0.86 ± 0.12	—	0.88 ± 0.14	NS
	FN Z-score	−0.81 ± 1.18	—	−0.66 ± 1.25	NS
	FN T-score	−1.12 ± 1.25	—	−0.91 ± 1.29	NS
De Rosa et al 2001 (25)	LS BMD (g/cm^2^)	0.804 ± 0.04	1.080 ± 0.01	0.978 ± 0.05	<.001*^b^* < .05*^c^*
	LS Z-score	−2.59 ± 0.34	−0.06 ± 0.09	−0.97 ± 0.41	NR
	LS T-score	−2.60 ± 0.35	−0.07 ± 0.09	−1.02 ± 0.42	NR
	FN BMD (g/cm^2^)	0.850 ± 0.01	0.948 ± 0.02	0.892 ± 0.03	NS
	FN Z-score	−0.81 ± 0.11	+0.14 ± 0.25	−0.41 ± 0.37	NR
	FN T-score	−0.77 ± 0.11	+0.12 ± 0.22	−0.39 ± 0.31	NR
Ozisik et al 2001 (38)	Vertebral BMD (g/cm^2^)	0.77 ± 0.029	1.190 ± 0.013	—	<.001
Leifke et al 1998 (36)	Trabecular BMD (mg/cm^3^)	congenital: 82 ± 10 acquired:112 ± −20	—	Klinefelter: 125 ± 12 acquired: 117 ± 13	NR
	Cortical BMD (mg/cm^3^)	congenital: 221 ± 27 acquired: 255 ± 23	—	Klinefelter: 276 ± 31 acquired: 268 ± 7	NR
Lubushitzky et al 1998 (37)	LS BMD (g/cm^2^)	0.93 ± 0.09	1.07 ± 0.15	—	<.009
	FN BMD (g/cm^2^)	0.95 ± 0.11	1.08 ± 0.16	—	<.006
	LS QBS (%ID/mL × 10^−3^)	5.43 ± 1.58	5.50 ± 1.15	—	NS
	FN QBS (%ID/mL × 10^−3^)	4.19 ± 1.86	3.81 ± 1.28	—	NS
Behre et al 1997 (31)	BMD using qCT on lumbar spine (mg/cm^3^ hydroxyapatite)	84.1 ± 6.5	—	105.0 ± 9.1	NR
Choi et al 1995 (33)	LS Z-score	−0.37 ± 0.30	—	−0.78 ± 0.19	NS
	FN Z-score	−0.20 ± 0.37	—	−0.24 ± 0.18	NS
	Ward triangle Z-score	0.39 ± 0.39	—	0.33 ± 0.24	NS
Finkelstein et al 1989 (41)	Cortical (measured using?) bone density (g/cm^2^) in those with fused epiphyses	0.74 ± 0.01	0.86 ± 0.01	—	<.001
	Cortical bone density (g/cm^2^) in those with open epiphyses	0.70 ± 0.03	0.79 ± 0.02	—	< .001
	Trabecular bone density (mg/cm^3^)	116 ± 6	177 ± 4	—	<.0001
Finkelstein et al 1987 (42)	Cortical bone density (g/cm^2^) in those with fused epiphyses	0.71 ± 0.02	0.86 ± 0.01	—	<.0001
	Cortical bone density (g/cm^2^) in those with open epiphyses	0.61 ± 0.01	0.79 ± 0.02	—	<.001
	Trabecular bone density (mg/cm^3^)	112 ± 7	177 ± 4	—	<.0001

Abbreviations: BMD, bone mineral density; FN, femoral neck; HH, hypogonadotropic hypogonadism; LS, lumbar spine; NR, not reported; NS, not significant; PH, primary hypogonadism; TH, total hip; QBS, quantitative bone SPECT; qCT, quantitative computed tomography.

^*a*^When more than one set of data were reported in cohort studies, the latest results were included.

^*b*^Between HH and healthy control.

^*c*^Between HH and PH.

**Figure 4.**
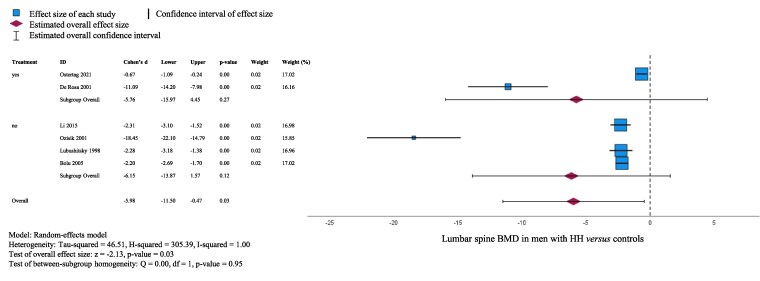
Meta-analysis of lumbar spine bone mineral density (BMD) in men with hypogonadotropic hypogonadism (HH) compared to healthy controls. Of the 6 studies, 2 were of men treated hormonally and the other 4 compared untreated men to healthy controls.

We performed a meta-analysis comparing LS BMD in men with HH and healthy controls in studies with mean and SD available (Fig. 4). Four studies were not included in the meta-analysis for the following reasons: not reporting BMD values (34); not reporting SDs and inability to calculate SDs due to nonnormal distribution (16); only reporting cortical and trabecular bone density (41, 42). As expected, LS BMD was lower in men with HH compared to healthy controls with a standardized mean difference (SMD) of −5.98 (95% CI, −11.5 to −0.47) (*P* = .03). However, subgroup analyses revealed no statistically significant difference in men with treated (*P* = .27) or untreated HH (*P* = .12) when compared with healthy eugonadal men. FN BMD comparisons were available only for 4 studies, so a meta-analysis was not conducted.

The largest study comparing men with HH to healthy controls was a cross-sectional study from France (51 men with CHH, 40 healthy eugonadal men) (13). Despite an average duration of hormonal treatment of 10.5 ± 6.9 years, areal BMD in LS, TH, and FN and distal radius and cortical and trabecular volumetric BMD were lower in men with CHH compared to healthy eugonadal men (13). The authors also reported impaired trabecular microarchitecture in men with HH compared with controls after 10.5 years of treatment. Higher trabecular bone volume and volumetric BMD were observed in men with CHH who started hormonal treatment at younger than 19 years and those with partial CHH, compared with other men with CHH.

In summary, BMD is lower in men with HH compared to healthy controls. However, it remains uncertain whether BMD completely normalizes compared with the general population following hormonal treatment.

### Comparing Bone Mineral Density in Men With Hypogonadotropic Hypogonadism Compared With Men With Primary Hypogonadism

Bone outcomes in men with HH were compared to men with primary hypogonadism (PH) in 8 studies (11, 16, 25, 27, 30, 31, 33, 36). Most studies included men either treated or untreated with hormonal treatment. Two studies included only men with Klinefelter syndrome in the PH group of men (25, 33), while 4 studies had a mixture of patients with PH due to various specified etiologies (11, 30, 31, 36). Two studies did not report the etiology of PH in their participants (16, 27). Results of the studies comparing men with HH to men with PH are summarized in Table 1. In the study by Giovanelli et al (11), the HH population included 5 of 47 men with functional hypogonadism; however, the summary statistics for all 47 were considered. Overall, 5 of 8 studies reported lower BMD in men with HH compared to PH (16, 25, 27, 31, 33). In some of these studies, men with HH had lower baseline total testosterone when compared with men with PH (25, 31, 33), but other studies did not report baseline TT concentration (16, 27). One of the 8 studies reported higher BMD in men with HH (LS Z-score, −0.7 ± 1.1) compared with PH (LS Z-score, −1 ± 1.4) (11). This is despite lower 25-OH D3 levels in men with HH (22.4 ± 12.7 ng/mL) compared with men with PH (27.8 ± 12.2 ng/mL). Two remaining studies did not observe a statistically significant difference in bone outcomes in men with HH compared with men with PH (30, 36).

In summary, some studies have observed that men with CHH have lower BMD compared with PH; however, confounders preclude our ability to accurately compare bone outcomes between the patient groups.

### Bone Mineral Density Changes in Men With Hypogonadotropic Hypogonadism With Reproductive Hormone Treatment

Several cohort studies have reported bone density changes following hormonal treatment in treatment-naive men (10, 14, 18, 19, 23, 24, 33, 36, 37), while other studies compared treated men with HH vs different men with untreated HH (10, 12, 14, 16).

A retrospective study of men with CHH reported cross-sectional data comparing 27 treated (median treatment duration: 7 years) with 42 untreated men (14). The authors observed significantly higher raw BMD and Z-scores in treated compared with untreated men with CHH (LS BMD; 0.89 ± 0.14 g/cm^2^ vs 1.14 ± 0.14 g/cm^2^; *P* < .001; FN BMD; 0.81 ± 0.12 g/cm^2^ vs 0.96 ± 0.13 g/cm^2^; *P* < .001; TH BMD; 0.77 ± 0.13 g/cm^2^ vs 0.96 ± 0.15 g/cm^2^; *P* < .001). The same retrospective study reported BMD change with treatment in 53 men with CHH with a variable duration of follow-up; however, only 6 men were followed up for more than 6 years. A significant increase in LS BMD (0.12 ± 0.08 g/cm^2^), but not in FN (0.07 ± 0.07 g/cm^2^), and TH (0.07 ± 0.06 g/cm^2^) was observed following treatment in men with CHH.

Antonio et al (10) compared T-score in treated (n = 19) vs treatment-naive (n = 6) men with CHH, and the change in Z-score during treatment in men with CHH for a median period of 11 years. T-score < −1 in the LS was observed in 61% men with CHH despite long-term treatment though the T-score improved during treatment (T-score improvement: LS; 2.19 ± 0.31; *P* < .01; FN; 1.47 ± 0.29; *P* < .001).

Results of all included studies reporting pretreatment and posttreatment bone outcomes in men with HH are summarized in Table 2. Improvements in at least one bone outcome were observed in all 8 studies; however, 1 study reported improvements only in the LS T-score, with no changes observed in the TH or FN T-scores (16). Several studies have examined bone outcomes in men with treated and untreated HH (Table 3), with most, but not all, demonstrating improvements in bone outcomes in treated men compared to those who were untreated.

**Table 2.** Summary of results of the studies comparing bone outcomes pre treatment and post treatment in the same men with hypogonadotropic hypogonadism

Study ID	Outcomes	Pre treatment	Post treatment	Significance
Antonio et al 2019 (10)	LS T-score	−3.59 ± 0.58	−1.4 ± 0.83	<.001
	TH T-score	−2.03 ± 0.59	−0.55 ± 0.75	<.01
	LS T-score < −1	6/6	3/6	NS*^a^*
	TH T-score < −1	5/6	2/6	NS*^a^*
Pazderska et al 2018 (19)	LS T-score	−3.35 ± 0.52	−2.057 ± 0.8	.0061*^a^*
	TH T-score	−2.21 ± 0.75	−1.64 ± 0.72	NS*^a^*
	FN T-score	−2.37 ± 0.8	−2.0 ± 0.93	NS*^a^*
Li et al 2015 (18)	LS BMD (g/cm^2^)	0.905 ± 0.153	1.017 ± 0.14	.002
	FN BMD (g/cm^2^)	0.802 ± 0.111	0.923 ± 0.10	.003
	TH BMD (g/cm^2^)	0.838 ± 0.123	0.920 ± 0.12	.003
Tirabassi et al 2014 (24)	LS BMD (g/cm^2^)	1.11 ± 0.05	1.16 ± 0.05	<.001
	Left FN BMD (g/cm^2^)	0.95 ± 0.05	0.97 ± 0.05	<.001
	Left trochanter BMD (g/cm^2^)	0.78 ± 0.01	0.81 ± 0.02	<.001
	LS T-score	0.71 ± 0.46	1.15 ± 0.45	<.001
	Left FN T-score	0.35 ± 0.42	0.46 ± 0.39	<.001
	Left trochanter T-score	−0.07 ± 0.15	0.15 ± 0.17	<.001
Lee et al 2014 (23)	LS BMD (g/m^2^)	1.067 ± 0.155	1.116 ± 0.177	.028
	FN BMD (g/m^2^)	0.908 ± 0.148	0.875 ± 0.212	NS
	Total femur BMD (g/m^2^)	0.968 ± 0.155	0.990 ± 0.166	NS
Lubushitzky et al 1998 (37)	LS BMD (g/cm^2^)	0.82 ± 0.04	0.93 ± 0.09	*P* < .001
	FN BMD (g/cm^2^)	0.87 ± 0.10	0.95 ± 0.11	*P* < .001
	LS QBS (%ID/mL × 10^−3^)	6.20 ± 2.47	5. 43 ± 1.58	NS
	FN QBS (%ID/mL × 10^−3^)	4.56 ± 2.13	4.19 ± 1.86	NS
Zhang et al 2021 (pre-53, post 30), median, IQR (14)	LS BMD (g/cm^2^)	0.921 (0.807-1.023)	1.021 (0.882-1.171)	.02*^a^*
	FN BMD (g/cm^2^)	0.812 (0.731-0.894)	0.877 (0.827-0.978)	.0026*^a^*
	TH BMD (g/cm^2^)	0.797 (0.705-0.896)	0.896 (0.787-0.966)	.003*^a^*
Choi et al 1995 (33)	LS Z-score	−1.68 ± 0.4	−0.37 ± 0.30	*P* < .01
	FN Z-score	−1 ± 0.43	−0.20 ± 0.37	*P* < .01
	Ward triangle Z-score	−0.32 ± 0.43	0.39 ± 0.39	*P* < .01

Abbreviations: BMD, bone mineral density; FN, femoral neck; IQR, interquartile range; LS, lumbar spine; NR, not reported; NS, not significant; TH, total hip; QBS, quantitative bone SPECT.

^*a*^ *P* values were not reported in the original study and were derived by the authors using the available summary data.

**Table 3.** Summary of results of the studies comparing bone outcomes in men with treated hypogonadotropic hypogonadism vs untreated hypogonadotropic hypogonadism

Study ID	Outcomes	Untreated men	Treated men	Significance
Antonio et al 2019 (10)	LS T-score	−3.59 ± 0.58	−1.94 ± 0.33	.0001*^a^*
	TH T-score	−2.03 ± 0.59	−0.93 ± 0.29	.0001*^a^*
	LS T-score < −1	6/6	13/17	NS*^a^*
	TH T-score < −1	5/6	9/17	NS*^a^*
Maione et al 2018 (12)	LS BMD (g/cm^2^)	1.00 ± 0.19	1.04 ± 0.19	NS*^a^*
	FN BMD (g/cm^2^)	0.86 ± 0.13	0.89 ± 1.16	NS*^a^*
	LS Z-score	−1.28 ± 1.80	1.14 ± 1.67	.77
	FN Z-score	−0.81 ± 0.98	−0.98 ± 1.22	.56
Gioia et al 2014 (16)	LS BMD (g/cm^2^)	1.05 (0.9 to 1.13)	1.18 (1.1 to 1.26)	NS
	LS T-score	−1.3 (−2.3 to −0.7)	0.1 (−0.1 to 0.3)	NS
	LS Z-score	−1.2 (−3.0; −0.2)	0.25 (−0.1 to 0.6)	NS
	FN BMD (g/cm^2^)	0.85 (0.73 to 0.94)	0.92 (0.86 to 0.98)	NS
	FN T-score	−1.6 (−2.3 to −1.0)	−0.6 (−0.7 to −0.5)	<.05
	FN Z-score	−1.05 (−1.8 to −0.4)	0.2 (0.0 to 0.4)	<.05
	TH BMD	0.96 (0.81 to 1.02)	1.06 (0.9 to 1.21)	NS
	TH T-score	−0.9 (−1.6 to −0.4)	0.0 (−0.9 to 0.9)	NS
	TH Z-score	−0.45 (−1.6 to 0.1)	0.5 (−0.7 to 1.7)	NS
Zhang et al 2021 (14)	LS BMD (g/cm^2^)	0.888 ± 0.135	1.138 ± 0.138	<.001
	FN BMD (g/cm^2^)	0.809 ± 0.116	0.960 ± 0.129	<.001
	TH BMD (g/cm^2^)	0.773 ± 0.126	0.961 ± 0.154	<.001
	LS Z-score	−2.174 ± 0.972	−0.328 ± 1.201	<.001
	FN Z-score	−1.524 ± 0.786	−0.345 ± 0.910	<.001
	TH Z-score	−1.774 ± 0.889	−0.237 ± 1.085	<.001

Abbreviations: BMD, bone mineral density; FN, femoral neck; LS, lumbar spine; NR, not reported; NS, not significant; TH, total hip; QBS, quantitative bone SPEC.

^*a*^ *P* values were not reported in the original study and were derived by the authors using the available summary data.

### Factors Associated With Bone Mineral Density in Men with Hypogonadotropic Hypogonadism

Age of men with HH at treatment initiation was the most frequently identified factor associated with BMD. Four studies observed higher BMD in those starting hormonal treatment earlier in life (13, 14, 25, 34). Other factors reported to be associated with better BMD were partial HH (13); duration of hormonal treatment (14); and free testosterone and estradiol concentrations (34). However, some studies failed to observe any association between reproductive hormone concentrations and BMD (14, 23, 42). Several studies were excluded because they did not provide data specific for men with HH rather than other causes of hypogonadism (11, 20, 22, 26-28, 30, 31, 33, 36).

### Fracture Outcomes in Men with Hypogonadotropic Hypogonadism

Only 2 studies have comprehensively assessed fracture prevalence as a study outcome. Giovanelli et al (11) assessed vertebral fractures by morphometric VF assessment (VFA) and other fragility fractures through medical history. They observed that 17% (8/47) men with HH (age 46.3 ± 12.5 years) had vertebral fragility fractures and 4.3% (2/47) had other fragility fractures. The authors observed no significant difference in fractures prevalence between men with HH and PH. Another study observed that in men with Kallmann syndrome (age 30.31 ± 9.38 years), 4 of 17 (23.5%) treated for less than 6 months with testosterone/human chorionic gonadotropin and 2 of 15 (13.3%) treated for more than 2 years had a history of fragility fractures. Furthermore, mild or moderate vertebral deformities (by Genant staging) were detected by VFA in 5 of 17 patients treated for less than 6 months (17). An additional 8 studies reported on fracture outcomes without formal fracture assessments (10, 12, 13, 15, 16, 18, 23, 42). A summary of studies reporting on fractures in men with HH is shown in Table 4. In summary, studies with systematic assessment for fractures as outcomes report relatively high fracture prevalence. However, studies without systematic fracture assessment fail to observe an increased fracture risk.

**Table 4.** Summary of the fracture outcomes reported by studies

Study ID	Bone outcomes reported	Men with HH group	Comparator group
Giovanelli et al 2022^*a*^ (11)			Men with PH
	Vertebral fractures	8/47	14/72
	Other fragility fractures	2/47	2/72
Iolascon et al 2015^*a*^ (17)	Fragility fractures	TRT < 6 mo 4/17 TRT > 6 mo 2/15	None
	Vertebral deformities	TRT <6 mo 5/17	
Finkelstein et al 1987 (42)	Vertebral fracture	1/23	NR
	Loss of vertebral height (no distinct fracture)	2/23	NR
Ostertag et al 2021 (13)	Fragility fractures	7/51	0 (based on inclusion criteria)
Antonio et al 2019 (10)	Fragility fractures	3/23	None
Maione et al 2018 (12)	Pathological fractures	0/31	None
Dutta et al 2016 (15)	Fragility fracture	1/22	Men with other endocrinopathies Graves disease 2/21 T1DM 2/30 PHPT 2/9 Andropause 8/44
Li et al 2025 (18)	Fracture	0/22	Age- and sex-matched healthy controls 0/20
Gioia et al 2014 (16)	Vertebral fracture	0/10	NR
Lee et al 2014 (23)	Clinical fracture	0/21	None

Abbreviations: HH, hypogonadotropic hypogonadism; NR, not reported; PH, primary hypogonadism; PHPT, primary hyperparathyroidism; T1DM, type 1 diabetes mellitus; TRT, testosterone replacement therapy.

^*a*^Fracture prevalence formally assessed as a study outcome.

## Discussion

Our systematic review confirms lower BMD, Z-scores, and T-scores in men with HH compared to healthy controls, and an improvement in BMD with subsequent hormonal treatment. Although these are expected observations, our meta-analysis suggests lower LS and FN Z-scores across various treatment durations indicating persistence of BMD below age- and sex-matched population values despite treatment. This is concerning since it would be assumed that testosterone treatment would normalize bone outcomes in men with HH. Unexpectedly, when evaluating HH subgroups, we observed men with acquired HH had a slightly higher pooled mean LS and FN Z-score; however, we anticipate this is likely due to random variation, rather than attributable to a true clinical phenomenon. Notably, not all studies included standardized management with vitamin D and calcium supplementation, which may have confounded some of the results.

Our extensive search on multiple databases and robust study selection process ensured the identification of eligible studies. Using 2 predefined data extraction tools depending on the study design and the National Institutes of Health quality assessment tool allowed objective assessment of the studies. However, we needed to exclude some studies in detailed synthesis due to the lack of data for men with HH alone. In addition, the wide heterogeneity of the study population (eg, congenital and acquired, treated and untreated) and the reported outcomes (areal BMD, volumetric BMD, Z-score, and T-score) limited synthesis of our results. We partly overcame this by using tailored data synthesis methods. For example, irrespective of the presence of a control group, we used Z-scores/T-scores to obtain an understanding of the BMD compared to population normative data. We also acknowledge there was a lack of high-quality studies, with 29 of the 33 included studies classified as “fair” or “poor” during quality assessment.

Sex steroids play a crucial role in bone turnover through estrogen and androgen receptors (3, 45). Therefore, low BMD is commonly observed in untreated men with HH. Similarly, testosterone treatment is expected to improve BMD. However, our findings question whether this oversimplifies a potentially complex scenario whereby low BMD might persist despite years of hormone therapy. This could be due to multiple reasons. One potential mechanism particularly related to CHH is missing the window of opportunity during the pubertal growth spurt and accrual of peak bone mass. There is evidence that late puberty can reduce the peak bone mass accrual despite some catchup (46). Poor adherence to treatment is reported among patients with CHH, possibly contributing to poorer real-life outcomes (47). Failure to achieve adequate sustained physiological levels of testosterone concentrations has been suggested in some of the studies as another potential mechanism (12). In addition, short-acting testosterone injections are known to be associated with fluctuations of testosterone concentration, which might expose men to subtherapeutic levels (48). The quality of TRT offered to men was not known and therefore could not be evaluated. It is conceivable that men with HH treated with adequate TRT from a young age would see improved bone outcomes; however it is not possible to conclude that with this study. Further studies would be needed to determine whether these factors or others would modify the BMD achieved in these patients. There might be other nonandrogen products from testes such as insulin like 3 (INSL3) involved in bone metabolism that are not corrected in standard reproductive hormone replacement (49). Furthermore, several mutations underlying CHH such as FGF8, FGFR1, and SEMA3A can have direct implications on bone health and therefore may confound the results (50).

Though we could not perform a meta-analysis, there was a trend toward lower BMD in men with HH compared with PH. PH groups mostly contained men with Klinefelter syndrome, and it is established that most adolescents with Klinefelter syndrome undergo some degree of puberty and retain some Leydig cell function (2). Hence more pronounced androgen deficiency in HH compared with PH as noted in several studies might be a confounder. We were unable to perform a subgroup comparison according to etiology of PH due to limited reporting of this in the included papers. Giovanelli et al (11), in contrast, reported lower BMD in PH, which is predominantly due to lower LS BMD in men with Klinefelter syndrome, while FN BMD was similar in men with other causes of PH and men with HH. In adult men, estradiol plays a regulatory role in bone homeostasis. Evidence suggests that serum concentrations as low as 35 pmol/L may be sufficient to prevent increased bone resorption and decline in BMD (51), and higher estradiol levels are associated with higher BMD (34). It is worth considering that different forms of male hypogonadism may be associated with varying levels of circulating estradiol, although this was not examined in this analysis. There is some evidence that there is a direct negative effect of FSH on bone metabolism, particularly trabecular bone (52). Therefore, current evidence is insufficient to resolve whether bone health differs between these two groups.

The most clinically relevant outcome in bone health is fractures. Since sex steroid effects are closely related to trabecular bone health, it is prudent to assess vertebral fractures in men with hypogonadism (5). Since up to two-thirds of vertebral fractures are clinically silent, vertebral fractures should be looked for radiologically (53). However, most of the studies did not include fracture as a separate clinical outcome or assessed fractures only from the history. Of studies that performed VFA, vertebral fractures were observed in 17% in one study (11) and another study found vertebral deformities in 29.4% of men with HH treated for less than 6 months with testosterone (17). In the same study, a history of fragility fracture was present in 18.8% of the men with HH. Though these studies did not have a healthy control group, the reported numbers are concerning for a young male population. Further interest in the ability of testosterone treatment to protect bone health in men has emerged with the surprising finding of increased fracture prevalence in men treated with testosterone in the Testosterone Replacement Therapy for Assessment of Long-term Vascular Events and Efficacy Response in Hypogonadal Men (TRAVERSE) study (6). While these data from middle-aged and older men with functional hypogonadism cannot be extrapolated to men with organic HH, these findings collectively call for closer attention to bone health in men with hypogonadism.

### Conclusion

Our systematic review and meta-analysis confirm that men with HH have low BMD compared to healthy controls. There is evidence that BMD improves with hormonal treatment although there is a suggestion that BMD remains below population norms despite long-term treatment. The small number of men in the studies with long treatment duration and the lack of high-quality evidence from the available observational studies particularly on fracture outcomes necessitate caution in interpreting these results and highlights the need for large, longer-term follow-up studies. This is crucial when also considering that fracture risk rises steeply with age. A multicenter cohort study with adequate follow-up would be the way forward to resolve these doubts.

## Data Availability

Some or all data sets generated during and/or analyzed during the current study are not publicly available but are available from the corresponding author on reasonable request.

## Abbreviations

BMD: bone mineral density
CHH: congenital hypogonadotropic hypogonadism
FN: femoral neck
HH: hypogonadotropic hypogonadism
LS: lumbar spine
PH: primary hypogonadism
RCT: randomized controlled study
ROB: risk of bias
TH: total hip
TRT: testosterone replacement therapy
VFA: vertebral fracture assessment
